# Availability of Emergency Department Wait Times Information: A Patient-Centered Needs Assessment

**DOI:** 10.1155/2021/8883933

**Published:** 2021-04-22

**Authors:** Samantha Calder-Sprackman, Edmund S. H. Kwok, Renee Bradley, Jeffrey Landreville, Jeffrey J. Perry, Lisa A. Calder

**Affiliations:** Department of Emergency Medicine, University of Ottawa, Ottawa, Ontario, Canada

## Abstract

**Introduction:**

Many Emergency Departments (ED) publish wait times; however, the patient perspective in what information is requested and the quantity of information to post is limited.

**Methods:**

We conducted a mixed-methods study at a tertiary care academic center. First, we conducted focus groups of 7 patients. We then generated themes following content analysis to create a patient survey. We administered in-person surveys to patients in ED waiting rooms at sites randomized for survey administration. We used preassigned shifts utilized for even patient perspective representation of the 24 hours-a-day/7 days-a-week service. We included waiting room patients over 18 years of age and excluded patients directly referred to a specialty service or who did not speak French or English. We analyzed survey data using descriptive statistics.

**Results:**

We identified nine dominant focus group themes: wait time definition, wait time notification, communication, education, patient expectations, utilization of the ED, patient behaviour, physical comfort, and patient empowerment. Of the 240 patient questionnaires administered, 81.3% of respondents wanted to know ED wait times before hospital arrival hospital and 90.8% wanted ED wait times posted in the waiting room. Website (46.7%) was the most popular choice for publishing wait times outside the ED. Within the ED, patients had no preference regarding display modality, if times were displayed (39.6%). Overall, 76.7% stated that their satisfaction with the ED would be improved if wait times were posted.

**Conclusion:**

ED patients strongly supported having access to wait time information. Patients believed having wait time information will have a positive impact on their overall ED satisfaction.

## 1. Introduction

Posting Emergency Department (ED) wait times stands out as a contentious topic. Many EDs rationalize publishing wait times as a strategy to improve patient satisfaction and decrease wait times by deflecting patient volumes from congested EDs [1, 2]. However, it has also been argued that there is little evidence that online publication of ED wait times decreases crowding. Critiques of posting ED wait times express concern about a theoretical risk of patients with serious medical conditions self-triaging to avoid the ED due to long wait times. Others have argued that self-triaging is safe as patients have a reasonable ability to predict the severity of their illness [3, 4].

Uncertainty exists around what quantitative metrics should be posted on ED wait time websites. Although transparency of ED performance may benefit patients, some patients may not have the knowledge of common ED functions such as triage [5]. While research has debated whether or not patients will use posted wait time information, limited research has sought to consider the patient perspective in what quality and quantity of information to post [2].

Patients are the consumers of health care and are important stakeholders in the Emergency Department (ED) waiting experience. The Institute of Medicine includes patient-centeredness as one of six aims of a high-quality health system [6]. When implementing new care models for patients, the patient perspective is critical [7]. The objective of this study was to describe and develop an understanding of the information needs of patients in the ED waiting room with respect to ED wait time notification.

## 2. Methods

### 2.1. Study Design and Setting

We conducted a mixed-methods study at a Canadian tertiary care academic center with two tertiary care academic EDs. Stage 1 consisted of patient focus groups and the development of a survey based on focus group results. Stage 2 involved the administration of our survey to the ambulatory population of ED patients. The Ottawa Health Sciences Network Research Ethics Board approved the study protocol and the study was executed from April 2015 to June 2016.

### 2.2. Focus Group Participants

The study was conducted in English and in French. We held two focus group discussions comprised of a total of seven individuals. Patients over the age of 18 with previous ED wait time experience were selected by the Ottawa Hospital Patient Advocacy Committee Coordinator using convenience sampling. Patients were selected if they had experience with the hospital ED and had previously contacted the Patient Advocacy Department with a concern regarding the wait time process. Participation in the focus groups was voluntary and confidential and we obtained written informed consent prior to starting the focus groups.

### 2.3. Focus Groups Data Collection

We used structured, planned prompts that were created by an investigator (Samantha Calder-Sprackman) and reviewed by two others (Edmund S. H. Kwok and Lisa A. Calder) to reduce facilitator bias in focus groups (see Supplementary Materials (Appendix 2) for Focus Group Question Guide). Efforts were made to ensure that participants were made to feel respected and psychologically safe. Prior to conducting focus groups, the 24 prompts were piloted for validity and reliability by two members of the public not involved in the study. Two focus groups were held in person with four and three participants, respectively. All focus group participants were over the age of 18 and had previously experienced the ED wait time process or had contacted the hospital Patient Advocacy Department with a concern regarding their ED wait time. The focus groups were facilitated by a bilingual facilitator (Samantha Calder-Sprackman) and participants were encouraged to speak in the language they were most comfortable (French or English). Each one-hour focus group was facilitated (Samantha Calder-Sprackman) and audio recorded by a single investigator (Samantha Calder-Sprackman). The audio recordings were then deidentified and transcribed verbatim into a text document (Samantha Calder-Sprackman). Data collection and analysis continued until thematic saturation was reached. [8].

### 2.4. Survey Data Collection

A 32-question patient survey was developed using the themes that emerged from the focus groups (see Supplementary Materials (Appendix 3) for Survey Questions). We elected to create our own survey as a review of the literature did not reveal any previously published surveys that adequately addressed our research question. Patient surveys were piloted among five patients in the ED waiting room for face and content validity. Based on this pilot, one question was removed. Survey questions were posed to patients by interviewers (Samantha Calder-Sprackman or Renee Bradley) and recorded on iPads during the time period from when patients were triaged until their disposition. ED sites for survey administration were randomized using a random number generator. Preassigned shifts for survey administration were also established to ensure an equal balance of surveys completed on weekdays, weekends, days (8h00-16h00), evenings (16h00-24h00), and nights (24h00-8h00) to ensure representation of the any different needs of patients that present at all times of the day.

### 2.5. Survey Participants

Patients over the age of 18 years were included in the surveys if they were in the ambulatory area of the ED waiting room. Patients were excluded if they were unable to answer the survey. Verbal informed consent was obtained prior to administering the survey from each participant. All available patients were approached during the study times.

### 2.6. Data Analysis

The focus group transcripts were analyzed using grounded theory. Investigators independently reviewed the transcripts using line-by-line coding and constant comparative analysis. Emerging concepts were coded and, through joint discussions between two investigators (Samantha Calder-Sprackman and Jeffrey Landreville), general themes were developed. Further discussions amongst the investigators (Samantha Calder-Sprackman, Jeffrey Landreville, Lisa A. Calder, and Edmund S. H. Kwok) led to the development of a final set of dominant themes. Surveys were analyzed using descriptive statistics.

## 3. Results

### 3.1. Focus Groups

Nine themes were identified from the focus groups, as displayed in Supplementary Materials (Appendix 1) with representative quotations. Thematic saturation was reached after the first focus group; the subsequent focus group did not identify new themes. The nine dominant focus group themes were (1) patient definition of wait time, (2) wait time notification method, (3) the lack of communication patients felt while waiting to be seen by physicians, (4) education regarding wait times and ED process in waiting room, (5) patient expectations in the ED waiting room, (6) how patients utilize the ED, (7) patient behavioural response to posting ED wait times, (8) physical comfort while waiting in the ED, and (9) patient empowerment during the ED wait.

### 3.2. Surveys

Overall, 240 surveys were completed by patients in the ED waiting room, 40 each on weekday and weekend day, evening, and night shifts. Overall, 42% of patients had 0 ED visits in the last year, while 47% had 1–5 visits and 11% had >5 visits. Patient respondent characteristics are shown in Table 1. Survey response rate was nearly complete with fewer than 10 people estimated to have refused (i.e., response rate >96%). The median age for study participants was 39 years and 56.3% of them (135/240) were female. Most patients had between 0 and 5 visits to the ED in the last year (89.2%, 214/240). The top 5 complaints for presentation to the ED were gastrointestinal (16.7%, 40/240), orthopedic (15.4%, 37/240), gynecological (14.0%, 33/240), cardiovascular (10.0%, 24/240), and neurological (9.2%, 22/240) complaints. Most of the patients surveyed were waiting for health care professional (nurse or physician) bedside assessment (44.6%, 107/240) during the time of survey administration. During times at which patients were surveyed, there were most often between 5 and 10 patients waiting to be seen in the ambulatory care treatment area (47.5%, 114/240) and most frequently the longest waiting time to be seen was between 1 and 4 hours (79.1%, 190/240).

When asked how patients define ED wait times, most survey respondents (62.9%, 151/240) defined ED wait time as “the time from when you arrive to hospital until the time you see the ED physician.” All other ED wait time definition options had under a 10% response. Patients felt that a reasonable wait time to see a physician would be less than 1 hour (32.9%, 79/240) or less than 2 hours (33.3%, 80/240). When asked to select all reasons why they came to the ED instead of to a primary care physician, 62.5% (150/240) felt that their problem was an emergency, which was best dealt with in the ED, 62.0% (149/240) thought they may need x-rays or blood work, 31.6% (76/240) indicated that there were no walk-in clinics open at the time of their presentation, 26.6% (64/240) indicated that they thought they might need admission to the hospital, and 21.7% (52/240) came to the ED because their family doctor was closed or on vacation.

When asked to select all the reasons why they felt they had to wait in the ED (patients could select more than one), the two most frequent answers were the following: (1) their complaint had been triaged as less urgent than others (61.3%, 147/240), or (2) the ED did not have enough nurses or doctors for the number of patients who need to be seen (57.5%, 138/240). A minority of patients felt that they did not know why they had to wait (13.3%, 32/240) or that they had to wait because the ED was not run efficiently (10.0%, 24/240).

Of the patients surveyed, 81.3% (195/240) wanted to know ED wait times before arrival to hospital and 90.4% (217/240) wanted wait time notification in the ED waiting room. Most patients also wanted information posted about what to expect during a typical ED visit (67.1%, 161/240). Patients indicated that ED wait time notification would impact their decision-making: 60.0% (144/240) stated they would go to the hospital with the shortest wait time and 19.2% (46/240) responded that they would be more likely to leave without seeing a physician if there was a long wait time as perceived by the patient. Furthermore, 86.3% (207/240) indicated that having access to ED wait times would allow them to better manage their other life commitments (e.g., childcare, work, and transportation).

**Figure 1:**
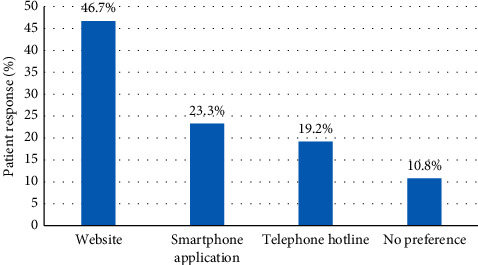
Patient number one ranked preference for modality of wait time notification prior to arrival in ED (*N* = 240).

**Figure 2:**
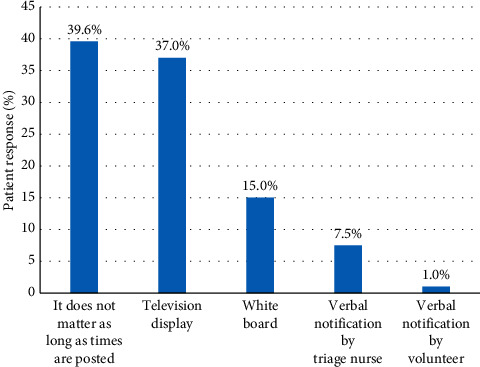
Patient number one ranked preference for modality of wait time publication in ED waiting room (*N* = 240).

Respondents ranked their top preference for modality of wait time notification prior to ED arrival and within the ED. Patients' top preference for modality of wait time notification prior to arrival was website (46.7%) (Figure 1), whereas, within the ED, patients were not particular about the specific modality if wait times were displayed (39.6%) (Figure 2). Displaying wait times using a digital monitor within the ED was also strongly endorsed (37.0%). Overall, 76.7% (184/240) stated that their satisfaction with the ED experience would be improved if wait times were posted either prior to arrival or in the ED.

## 4. Discussion

Patients surveyed in this study strongly supported having access to ED wait time information prior to arrival as well as in the ED waiting room. Most patients defined ED wait time as the time from triage to physician initial assessment and wanted the physician initial assessment time posted. Overall, patients felt that knowing ED wait times would allow them to better manage their personal commitments while they receive health care. Notably, over three-quarters of patients expressed that their overall satisfaction with the ED experience would be improved if ED wait times were posted.

ED crowding and long wait times are a challenge for EDs in Canada as in many other countries [9]. Waiting to see a physician can lead to patient stress, tense doctor-patient relationships, and patient dissatisfaction [10–13]. An important predictor of patients' satisfaction with their ED care is their perception of their wait time experience [10, 13–15]. Between 25 and 35% of patients in previous studies accurately predicted their physician initial assessment time since patients often overestimate their wait times in the ED [10, 15].

Although there is some evidence that longer wait times are generally associated with lower patient satisfaction [16, 17], Thompson found that actual wait times were not predictive of overall patient satisfaction and that patient perception of wait times being less than expected and satisfaction with information delivery in the ED were associated with higher overall satisfaction [10]. Kamali surveyed low-acuity patients in the ED waiting room and also found that 70% of respondents wanted a better estimate of wait times and explanation for reason for waiting. These findings coupled with the findings from our study demonstrate that transparency of information and updates regarding the wait process in the ED is important to patients. Improving information delivery regarding wait times is impactful on patient perception of their health care experience and may help achieve improvement in patient satisfaction experience.

A patient safety concern when notifying patients of ED wait times is that patients may use the information to self-triage and fail to present to the ED with a serious medical condition because of a long posted wait time [18, 19]. Yip surveyed 1,211 patients to investigate patients' awareness of online wait time information and willingness to use such information when choosing between two academic EDs in London, Ontario. [2] Survey results showed that 45% of respondents would use the available data to make a decision to choose an ED and that 44% would be more likely to go to the ED with a shorter wait time. In contrast, these findings are slightly more conservative than our patient survey respondents of whom 60.0% stated they would go to the hospital with the shortest wait time and 19.2% responded that they would be more likely to leave without seeing a physician if there was a long wait time. This is important for our field because it further highlights that many patients will use wait time information to self-triage to a specific ED.

A survey of 1,508 ED patients who left without being seen from two urban Canadian EDs found that although a long wait time was the most cited reason for patients leaving (79%), patients' concern about their medical condition was the most common reason for staying to see an ED physician (96%) [20]. Furthermore, Fraser found that one of the top responses for improving likelihood of staying in the ED to see a physician was providing patients more information on wait times (41%), which suggests that providing wait time information may be a way to alter tolerance for waiting and change the rate of patients leaving. [20] Another study by Guttmann et al. assessed the impact of ED wait times on the risk of adverse events including the outcomes of 617,011 patients who left without being seen from all EDs in Ontario, Canada, with mean annual patient volume above the 25% percentile [4]. Those who left the ED without being seen actually were not at any increased risk of short-term mortality or adverse event [4]. These results may indicate that patients have an appreciation when there is a high acuity medical concern and alter their behaviour accordingly [4, 20].

There is currently no universally accepted definition of what wait time information should be posted, and the majority of the systems in existence publish information deemed important from a provider perspective for marketing purposes and assisting with workload distribution between hospitals [19]. This study describes the quality and quantity of wait time information needs of patients in the ED waiting room. Our findings contribute important information that can be considered by administrators to develop a patient-centered approach for optimal delivery of wait time information to improve patient experience.

There are some limitations to our study. First, our study was conducted at a single academic center with two tertiary care sites and therefore may not be generalizable to other hospital EDs, especially outside of Canada, where the ED waiting room system and patient expectations may be different. Second, this study was conducted in a city where walk-in clinics post wait times online and therefore results from our study may not be generalizable to cities where this is not the case. Finally, survey respondents in our study were approached in the ED waiting room and asked if they wanted to participate in our study. We do not have statistics describing the general population presenting to the ED during the time of the study. The use of convenience sampling for survey administration may have created a risk of selection bias and social desirability bias. However, we tried to mitigate this by (1) randomizing site for survey administration and (2) creating shifts to ensure that equal number of patients were included to represent the 24 : 7 service provided. We did not record the total number of patients approached to complete surveys; however, under 10 patients refused to participate in our survey.

### 4.1. Future Directions

The results that have been generated from this study stimulate future lines of investigation. The first is to replicate and compare findings in other Canadian EDs across the country to demonstrate generalizability of results and subsequently create patient-centered recommendations for standardization of ED wait time notifications. Furthermore, an implementation study based on patients' identified needs from our study is required to further evaluate how publishing up-to-date, physician initial assessment wait time information affects patient satisfaction and experience in the ED.

## 5. Conclusions

ED patients strongly supported having access to wait time information. Patients believed that having wait time information will have a positive impact on their overall ED satisfaction.

## Figures and Tables

**Table 1:** Demographics and baseline characteristics of emergency department waiting room survey respondents (*N* = 240).

Characteristic	%, *N*^*∗*^
*Age (years)*	
Median, IQR	39.0, 24.0
Sex (M)	56.3, 135

*Top 5 chief complaints for presentation to ED*	
Gastrointestinal	16.7, 40
Orthopedic	15.4, 37
Gynecological	14.0, 34
Cardiovascular	10.0, 24
Neurological	9.2, 22

*Current stage of waiting*	
Waiting for nurse bedside assessment	44.6, 107
Waiting to see physician for the first time	11.6, 27
Waiting for tests	20.1, 49
Waiting for reassessment by physician	20.5, 50
Waiting to be seen by consulting service	3.2, 7

*Number of patients waiting to be seen in waiting room*	
<5	28.3, 68
5–10	47.5, 114
>10	24.2, 58

*Longest waiting to be seen time*	
<1 hour	8.8, 21
1–2 hours	22.5, 54
2–3 hours	28.3, 68
3–4 hours	28.8, 69
>4 hours	11.7, 28

*Patients' stated reasonable ED wait time*	
No wait time	4.0, 8
Less than 30 minutes	11.2, 26
Less than 1 hour	32.1, 79
Less than 2 hours	32.5, 80
Less than 3 hours	12.4, 29
As long as it takes to get the care I need	7.6, 18

*Where patients would go if they left ED without being seen*	
Another emergency department	48.6, 116
Walk in clinic	13.7, 33
Family doctor	5.4, 13
Home	22.1, 53
Return to same emergency department later	10.4, 25

^*∗*^%, *N*: unless otherwise specified.

## Data Availability

The data are available from the authors upon reasonable request.

## Supplementary Materials

Supplementary Materials Nine themes were identified from the focus groups, as displayed in Appendix 1 with representative quotations. Appendix 2 gives Focus Group Question Guide. A 32-question patient survey was developed using the themes that emerged from the focus groups (see Appendix 3 for Survey Questions).

## References

[1] Grafstein E. (2012). The waiting game: the emergency patient as a customer. *Canadian Journal of Emergency Medicine*.

[2] Yip A., Mcleod S., Mcrae A., Xie B. (2012). Administration Influence of publicly available online wait time data on emergency department choice in patients with noncritical complaints. *Canadian Journal of Emergency Medicine*.

[3] Innes G., Cooke T., Schorn R., Grafstein E., Rowe B. (2009). Are parental assessments of pediatric illness acuity accurate compared to nurse triage assessments?. *Canadian Journal of Emergency Medicine*.

[4] Guttmann A., Schull M. J., Vermeulen M. J., Stukel T. A. (2011). Association between waiting times and short term mortality and hospital admission after departure from emergency department: population based cohort study from Ontario, Canada. *British Medical Journal*.

[5] Seibert T., Veazey K., Leccese P., Druck J. (2014). What do patients want? survey of patient desires for education in an urban university hospital. *Western Journal of Emergency Medicine*.

[6] Health Quality Ontario (2015). *Quality Matters: Realizing Excellent Care For All*.

[7] Graff L., Stevens C., Spaite D., Foody J. (2002). Measuring and improving quality in emergency medicine. *Academic Emergency Medicine*.

[8] Morse J. M. (1995). The significance of saturation. *Qualitative Health Research*.

[9] Bond K., Ospina M., Blitz S. (2007). Frequency, determinants and impact of overcrowding in emergency departments in Canada: a national survey. *Healthcare Quarterly*.

[10] Thompson D. A., Yarnold P. R., Williams D. R., Adams S. L. (1996). Effects of actual waiting time, perceived waiting time, information delivery, and expressive quality on patient satisfaction in the Emergency Department. *Annals of Emergency Medicine*.

[11] Boudreaux E. D., O’Hea E. L. (2004). Patient satisfaction in the emergency department: a review of the literature and implications for practice. *The Journal of Emergency Medicine*.

[12] Soremekun O. A., Takayesu J. K., Bohan S. J. (2011). Framework for analyzing wait times and other factors that impact patient satisfaction in the emergency department. *The Journal of Emergency Medicine*.

[13] Toma G., Triner W., McNutt L.-A. (2009). Patient satisfaction as a function of emergency department previsit expectations. *Annals of Emergency Medicine*.

[14] Ackroyd-Stolarz S., Read Guernsey J., Mackinnon N. J., Kovacs G. (2011). The association between a prolonged stay in the emergency department and adverse events in older patients admitted to hospital: a retrospective cohort study. *BMJ Quality & Safety*.

[15] Thompson D. A., Yarnold P. R., Adams S. L., Spacone A. B. (1996). How accurate are waiting time perceptions of patients in the emergency department?. *Annals of Emergency Medicine*.

[16] Anderson R. T., Camacho F. T., Balkrishnan R. (2007). Willing to wait?: the influence of patient wait time on satisfaction with primary care. *BMC Health Serv Res*.

[17] Morgan M., Salzman J., LeFevere R., Thomas A., Isenberger K. (2015). Demographic, operational, and healthcare utilization factors associated with emergency department patient satisfaction. *Western Journal of Emergency Medicine*.

[18] Xie B., Youash S. (2011). The effects of publishing emergency department wait time on patient utilization patterns in a community with two emergency department sites: a retrospective, quasi-experiment design. *International Journal of Emergency Medicine*.

[19] ACEP (2012). *Publishing Wait Times for Emergency Department Care*.

[20] Fraser J., Atkinson P., Gedmintas A., Howlett M., McCloskey R., French J. (2017). A comparative study of patient characteristics, opinions, and outcomes, for patients who leave the emergency department before medical assessment. *CJEM*.

