# Supplementary material for: Availability of Emergency Department Wait Times Information: A Patient-Centered Needs Assessment
Source: Emerg Med Int. 2021 Apr 22;2021:8883933. doi: 10.1155/2021/8883933 (PMC8084678; doi:10.1155/2021/8883933)
Supplement: Supplementary Materials — Nine themes were identified from the focus groups, as displayed in Appendix 1 with representative quotations. Appendix 2 gives Focus Group Question Guide. A 32-question patient survey was developed using the themes that emerged from the focus groups (see Appendix 3 for Survey Questions). [file 8883933.f1.docx]

Appendix 1. Focus Group Themes (N=7)

| Definition of ED wait time | "I count the first waiting room and the hallway and the room I am led to until I see the doctor. So point of entry until I actually see a doctor." |
| --- | --- |
| Wait time notification | "It would be nice to know ahead of time that… I should call my grandmother or the aunt or the neighbor to go get my child from the … school daycare."  "… if they came in and a wait time was posted also that was updated real time when you come in would be good" |
| Lack of Communication | "Tell me if I’m going to have to wait 6 hours, I’ll go somewhere and have a coffee, or I’ll get someone to bring me a coffee."  “I do understand that there are emergencies coming in so that does cause some delay but there is no communication about the delay.” |
| Education in the ED waiting room | "That’s why it would be nice if the triage nurse just said that it’s going to be some time between 1-2 hours. Or it’s going to be about 4 hours for your care because she also knows where she is putting you in the line."  "I don’t know if they can do it digitally. They have the TVs on there… if there was some way to do it with the TVs on. " |
| Patient expectations | “I expect to wait varying lengths of time based on the reason for which I am at the Emerg…but I would hope that I have information from the staff. I wouldn’t expect to just sit there and have no information” |
| Patient behaviour | "I think if you are not feeling well and you are a responsible person, you are going to suck up the 6 hours or whatever you have to wait and you are going to grumble out and complain about it but you will go and you will do it because you want to make sure that you are looked after."  "I honestly think it could help decision making either way. If I had that information at the time that I was not feeling well and knew that someone would see me in two hours if things weren’t going well than it would have changed my mind [and come to the ED]" |
| Utilization of the ED | "... A lot of the patients that I speak to, don’t have family physicians so they are using this as a primary means of a family doctor’s office. Which again, they feel they are entitled to and why is there a wait. So they don’t understand why they have to wait either." |
| Physical comfort in the ED | "If I could just be laying down, I’d wait as long as you like! But when you are literally sitting trying not to puke…you know… than you become exhausted and you become angry…" |
| Patient empowerment | "I think it would be empowering for patients to know the steps. It’s almost as important as the wait time I would say." |

APPENDIX 2: FOCUS GROUP QUESTION GUIDE

1. Things to say at start:

a. I am here to learn about patients’ experiences and needs with regards to ED wait times.

b. You are the expert of your own experiences. There are no right or wrong answers so just do the best you can with the questions.

c. Feel free to ask for clarification at any point in time during the focus group.

1. Explain the GOAL of the study by saying the following and then move on to questioning:
   1. **The GOAL of this study is** to understand what patients want when it comes to publishing wait times for the emergency department.
      1. **Definition of the ED wait time**
         1. What does emergency department wait time mean to you?
      2. **Perceptions of ED wait times**
         1. What is your understanding of why you wait in the ED?
         2. Do you think that all patients wait the same amount of time regardless of presenting complaint?
         3. Do you think that there are good and bad times to come to the hospital with respect to wait times (e.g. 5am Sunday morning vs. 11pm Friday evening)?
      3. **Current experiences with ED wait times**
         1. What are your current experiences with ED wait times?
         2. Is there anything good about the ED wait time experience?
         3. What do you dislike about the ED wait time experience?
         4. Where are there opportunities to improve the ED wait time experience?
      4. **Publication of ED wait times**
         1. Would you like to have access to wait time information before arriving to the ED?
         2. Would you like to have access to wait time information after arriving to ED?
         3. Why would you like to know this information?
      5. **Medium for ED wait time publication**
         1. What would be your ideal medium for a method of ED publication of wait times? (e.g. telephone screen in the ED, piece of paper from triage/registration, orally from triage/registration, online website publication)
      6. **Influence on patient behaviour**
         1. How would you use the publication of ED wait times?
         2. If the wait times were published online, would you use it for decision making (e.g. not going to the ED at all or selecting a specific ED to attend)?
         3. Do you think there would there be an increase in the number of patients that leave without being seen if people knew how long they would have to wait to be seen? If so, where do you think they would go (e.g. other ED, walk in clinic, family doctor at a later date, no care at all)?
         4. Do you think the publication of ED wait times would improve patient satisfaction?
      7. **Influence of external factors**
         1. Are certain patients less likely to wait in the ED (e.g. those with young children, elderly relatives or pets to care for)?
         2. Are certain patients more likely to wait longer in the ED (e.g. retired individuals, homeless)?
         3. Does having primary care provider (e.g. family doctor) influences if a patient presents to the ED?
         4. How come people go to the ED? (e.g. because they think they need sutures, need an x-ray, or they want a referral to a specialist)
      8. **Specific information on ED wait times**
         1. Would your ED wait time experience be improved if you were explained the steps of the ED wait time experience?
         2. What is the most important and useful information you want to know about wait time? (e.g. average wait time, number of patients waiting in the waiting room, number of patients being seen per hour, average wait time for a select number of typical presenting symptoms such as “abdominal pain…2 hours; chest pain…1 hour”)
         3. How would you like this information displayed? (quantified with numerical values, red/yellow/green light, compared vs. monthly or yearly highs/lows to put it into context)
         4. Is there any information you think should not be publicized?
2. **Prompts:**
   1. How can you be sure of this?
   2. How do you know?
   3. What happens if this information is not known?
   4. Could you tell more about this?

APPENDIX 3: SURVEY QUESTIONS

**Development of a Patient-Centered Model for the Publication of Emergency Department Wait Times:**

**Patient Questionnaire**

**SURVEY ADMINISTRATION:**

Date (DD/MM/YYYY):

Time:

**DEMOGRAPHIC INFORMATION:**

Age:

Gender:

Number of previous trips to the Emergency Department in the last year (not including this one):

What is the main reason(s) for coming today?

1.

2.

3.

Current stage of waiting:

1. Waiting for RN bedside assessment
2. Waiting to see physician for the first time
3. Waiting for tests
4. Waiting for reassessment by an Emergency Department physician
5. Waiting to see a physician from another service (e.g. General Surgery, Orthopedic Surgery)

**WAIT TIME DEFINITION:**

*When answering the questions below, please consider the reason why you are in the Emergency Department today.*

1. **Please complete the following sentence based on what the phrase ‘Emergency Department wait time’ means to you? Chose only one answer.**

**Emergency Department wait time is the time from arrival to the hospital until…**

- 1. You are seen by a nurse who asks why you are at the Emergency Department today (triage).
  2. You get to the main waiting area.
  3. You are seen by a nurse for a bedside nursing assessment.
  4. You see Emergency Department physician.
  5. Tests (e.g. bloodwork)
  6. Treatment (e.g. pain control medication)
  7. You are admitted or discharged.

1. **What is your understanding of *why* you need to wait in the Emergency Department today?**

**Chose as many answers that apply.**

- 1. Because my complaint has been triaged as less urgent than others.
  2. Because The Ottawa Hospital is a teaching hospital and there are residents and medical students who are also providing patient care.
  3. Because there are patients in the Emergency Department who do not need to be here and could go to another health care provider.
  4. Because the Emergency Department does not have enough nurses and/or doctors for the number of patients needing to be seen.
  5. Because the Emergency Department is not run efficiently.
  6. Because there is not enough Emergency Department space to see patients.
  7. Because the beds in the hospital are full and therefore people in the beds in the Emergency Department cannot be moved elsewhere in the hospital.
  8. I don’t know why I have to wait.

1. **What is a reasonable Emergency Department wait time?**
   1. No wait time.
   2. Less than 30 minutes
   3. Less than 1 hour
   4. Less than 2 hours
   5. Less than 3 hours
   6. Whatever wait time it takes to see a physician and get the care I need.

**UTILIZATION OF THE EMERGENCY DEPARTMENT**

*When answering the questions below, please consider the reason why you are in the Emergency Department today.*

1. **Why did you come to the Emergency Department as opposed to a family doctor or walk in clinic? Please check all that apply.**
   1. I believe that my problem is an emergency which is best dealt with in an Emergency Department.
   2. I think I might need to be admitted to hospital.
   3. There are no Walk-in clinics open at this time.
   4. I do not have a family doctor.
   5. I think I need an Xray, bloodwork, or other tests.
   6. My family doctor is closed or on vacation.
   7. I was unable to get an appointment with my family doctor.
   8. I do not like my family doctor.

**POWER DIFFERENTIAL IN THE WAITING ROOM**

*When answering the questions below, please consider your visit in the Emergency Department today.*

1. **Do you feel comfortable approaching a member of the health care team (e.g. nurse, physician, volunteer) if you believe that you have become sicker while waiting in the waiting room?**
   1. Yes
   2. No
      1. *If no, why:*
2. **Would you like a member of the health care team to check on you after waiting in the Emergency Department if you have still not seen a doctor after two hours?**
   1. Yes
   2. No

**WAIT TIME POSTING:**

*When answering the questions below, please consider your visit in the Emergency Department today.*

1. **Would you like to know Emergency Department wait times before arriving at the hospital?**
   1. Yes
   2. No
2. ***If yes*, how would you like this information posted? Rank the following answers from 1 to 3 (most important to least important).**

__ Website

__ Smartphone App

__ Telephone Hotline (e.g. Telehealth, hospital phone number)

1. **If you had access to Emergency Department wait times for all hospitals in Ottawa beforehand, would you go to the hospital with the shortest wait time?**
   1. Yes
   2. No
   3. It depends

*Please explain:*

1. **Would you like to have Emergency Department wait times posted in the waiting room when you arrive at the hospital?**
   1. Yes
   2. No
2. ***If yes,* how would you like Emergency Department wait times posted in the waiting room?**
   1. White board
   2. Television display
   3. Told by the triage nurse
   4. Told by a volunteer in the waiting room
   5. It does not matter how they are posted, as long as they are posted
3. ***If yes,* what would be the best way for Emergency Department wait times to be quantified?**
   1. Absolute time in hours and minutes (e.g. 1h35mins)
   2. Range of times (e.g. 30 mins -1 hour, 1 hour -1.5 hours, etc)
   3. Red light/yellow light/green light (e.g. Red light = >2 hours, yellow light =1-2 hours, green light = 1 hour)
4. ***If yes,* how often should the posted wait times be updated?**
   1. Every 30 minutes
   2. Every hour
   3. Every hour and a half
   4. Every two hours
   5. It doesn’t matter as long as the last time it was updated is posted

**CHANGE IN PATIENT BEHAVIOR/PATIENT DECISION MAKING**

*When answering the questions below, please consider your visit in the Emergency Department today.*

1. **If you arrived today at the Emergency Department and saw a long wait time posted, would you be more likely to leave without being seen by a physician?**
   1. Yes
   2. No
   3. Only if I thought the reason why I came to the Emergency Department did not need immediate medical attention.
2. **Where would you go** **if you left the Emergency Department without being seen by a physician?**
   1. Another Emergency Department
   2. Walk in Clinic (e.g. Appletree clinic)
   3. Family Doctor
   4. Home
   5. I would come back to the same Emergency Department later

**PATIENT EXPECTATIONS:**

*When answering the questions below, please consider your visit in the Emergency Department today.*

1. **Would your satisfaction with your Emergency Department experience be improved if wait times were posted?**
   1. Yes
   2. No

**For the next two statements, please indicate if you agree or disagree.**

1. **As a patient, I would find the posting of Emergency Department wait times empowering.**
   1. Agree
   2. Disagree
2. **Having access to Emergency Department wait times prior to arrival, would allow me to better manage my other commitments (e.g. childcare, work, transportation).**
   1. Agree
   2. Disagree

**PATIENT EDUCATION IN THE WAITING ROOM/COMMUNICATION:**

*When answering the questions below, please consider your visit in the Emergency Department today.*

1. **Once arrived at the Emergency Department, would you like information posted on what to expect during a typical Emergency Department visit (e.g. waiting in waiting room, seen by nurse, seen by physician, waiting for blood work, waiting for reassessment)?**
   1. Yes
   2. No
2. **Would you like information on how to find a family doctor posted in the waiting room?**
   1. Yes
   2. No
